# Customizing the extracellular vesicles release and effect by strategizing surface functionalization of titanium

**DOI:** 10.1038/s41598-022-11475-3

**Published:** 2022-05-05

**Authors:** Miquel Antich-Rosselló, Maria Antònia Forteza-Genestra, Javier Calvo, Antoni Gayà, Marta Monjo, Joana Maria Ramis

**Affiliations:** 1 Cell Therapy and Tissue Engineering Group, Research Institute on Health Sciences (IUNICS), University of the Balearic Islands (UIB), Ctra. Valldemossa km 7.5, 07122 Palma, Spain; 2 Health Research Institute of the Balearic Islands (IdISBa), Palma, Spain; 3 Fundació Banc de Sang i Teixits de les Illes Balears (FBSTIB), Palma, Spain; 4 Departament de Biologia Fonamental i Ciències de la Salut, UIB, Palma, Spain

**Keywords:** Biochemistry, Biophysics, Biotechnology, Stem cells, Molecular medicine, Materials science, Nanoscience and technology

## Abstract

Metallic material functionalization with Extracellular Vesicles (EVs) is a desirable therapeutic approach to improve regenerative procedures. Among the different functionalization strategies available, here we have compared drop casting on machined Ti surfaces, drop casting on nanostructured TiO_2_ surfaces and polymeric entrapment with polydopamine. EVs are a heterogeneous population of communication nanovesicles released by cells that are being intensively investigated for their use in therapeutics. We have selected platelet derived EVs for Ti surface coating due to their demonstrated osteoinductive properties. Our results show that each functionalization strategy leads to differences in the size of EV populations attached to and released from the metallic implants, which, in turn, leads to variations in their osteogenic capability measured through alkaline phosphatase activity and calcium deposition. In conclusion, the functionalization strategy used has an important effect on the resulting implant functionality, probably due to the heterogeneous EVs nature. Thus, the methodological approach to metallic material functionalization should be carefully chosen when working with extracellular vesicles in order to obtain the desired therapeutic application.

## Introduction

Extracellular vesicles (EVs) are biological membranous nanoparticles released by all cell types, with sizes ranging from 30 nm to at least 5 µm^1^. There exist different EV subpopulations, which include exosomes, microvesicles or apoptotic bodies, and each of them vary on their biogenesis mechanism, composition, size or function^1^. Furthermore, EVs enable communication pathways between cells by allowing them to exchange different biomolecules—referred as cargo—such as proteins, lipids or nucleic acids^2^. Intensive investigation has shown that the administration of EVs alone offers comparable or even present alternative therapeutic opportunities than the use of stem cell therapy, which was once heralded as the miracle cure in regenerative medicine^3,4^.

In therapeutics, among all the different kinds of EVs, platelet derived EVs (pEVs) present a special interest for their therapeutical activity^5–7^, since platelets are widely known for their regenerative capability. Thus, platelet concentrates, such as platelet rich plasma (PRP) or platelet lysate (PL) have been used for musculoskeletal regeneration, osteoarthritis or complex wounds treatment^8–10^. Recently, pEVs have emerged as platelet actuators, which may drive, at least in part, their regenerative capability^5^.

We and others have already reported that pEVs derived from PL present an osteogenic effect on mesenchymal stem cells^11,12^. Moreover, pEVs have also shown the capability to avoid osteonecrosis by promoting osteoblast proliferation and avoiding their apoptosis^13^. These osteogenic capabilities have been associated to the growth factors and miRNA contained into pEVs^11,14^. However, despite all the evidence that corroborate the osteogenic applicability of pEVs, there is still a lack of translation into the bone healing clinical field. In addition, when thinking on the clinical translation, pEVs have some advantages compared to cell cultured derived EVs, mainly related to safety and regulatory concerns: (1) clinical-grade allogenic platelets are already obtained from whole-blood donations and (2) there is no need for ex vivo cell expansion, diminishing concerns over contamination or immunological response^6^.

Moreover, another important aspect of EVs is their potential use as drug delivery systems^15^. The possibility of using EVs as carriers is under evaluation and it would be a huge improvement in their therapeutical applications. Some of the benefits of EVs compared to other synthetic drug delivery systems include the intrinsic ability to cross biological barriers and the low immune reactivity^15^. The loading strategies for EVs can be divided in those which are performed during EV biogenesis and those which are performed after EVs isolation. Since pEVs are derived from human blood, their biogenesis cannot be manipulated. Thus, loading must be performed after isolation and may include simple incubation^16,17^, sonication^18^, freeze-thawing^17^ or electroporation^19^. With this in mind, countless therapeutic applications can be developed through the use of EVs alone or in combination with biomaterials.

Titanium-based biomaterials are widely used in clinical orthopedic and dental interventions, due to their biocompatibility, mechanical properties, and long-term stability^20^. However, titanium implants also present some limitations. For instance, the outer oxide layer may lead to poor osteointegration due to the formation of fibrous tissue between the bone and the implant^21^. Additionally, many implants may fail during surgery procedure due to infections and biofilm formation around them, leading to patient suffering and increasing the expenses of the health care system^22^. Thus, we hypothesized that a Ti surface functionalized with pEVs or modified pEVs would gain regenerative capacity plus any additional function gained by pEV modifications, directed towards different biomedical applications.

Thus, some studies have already reported different strategies for Ti functionalization with EVs derived from cell culture media, which included chemisorption approaches^23,24^ and physisorption approaches^23,25–28^. As we have explained, the use of cell cultured derived EVs may hinder a future clinical use, whereas pEVs could evade these limitations^6^. In addition, no direct comparison among different strategies was performed in the previous studies. Thus, here, we have compared different strategies for Ti surface functionalization with pEVs: (i) through drop casting onto machined Ti surfaces, (ii) through drop casting onto nanostructured Ti surfaces and (iii) through polymeric entrapment strategies using poli-dopamine, and we have evaluated their physic-chemical properties (surface morphology, wettability and presence of EVs), the pEVs release profile over time and their biological activity in vitro with mesenchymal stem cells derived from human umbilical cord (hUC-MSCs).

## Experimental section

### Platelet derived EVs isolation and characterization

Buffy coats were obtained from the IdISBa Biobank and their use was approved by its Ethics Committee (IB 1995/12 BIO). Platelet concentrates were obtained as previously described and stored at − 20 °C until EV isolation^12^. After size exclusion chromatography, EVs enriched fractions were obtained and characterized as previously reported^29^. Briefly, morphology was determined by transmission microscopy, size and concentration were determined through nanoparticle tracking analysis (NTA) and the presence of CD9 and CD63 were assessed through western blot (WB). pEVs characterization is reported in Supplementary Fig. I. pEVs were stored at − 80 °C until use.

### Ti implant functionalization

Titanium discs (Ti), c.p. grade IV, 6.2 mm diameter and 2 mm height were purchased from Implantmedia (Lloseta, Spain). Implants were washed as previously described^30^, before their functionalization. Then, discs were functionalized following different strategies, which included drop casting (i), drop casting on nanostructured surfaces (ii) and polymeric entrapment (iii). (i) For drop casting strategies, Ti discs were first passivated with 30% HNO_3_ for 30 min, rinsed with water until the pH became neutral and left in water for 48 h. (ii) For surface nanostructuration, Nanonets (NN) nanostructure were produced on polished Ti implants as previously described^31^. Briefly, NN were electrochemically produced with an Autolab (Metrohm Autolab BV, Utrecht, The Netherlands) and then passivated by leaving them in water for 48 h. (iii) For polymeric entrapment, Ti discs were passivated with 30% HNO_3_ for 30 min, rinsed with water until the pH became neutral and left in water for 48 h. Then, a 4 mg/ml dopamine (Sigma, St. Louis, Missouri, USA) solution was prepared in a 10 mM Tris–HCl (Sigma) solution and brought to a pH of 8.5. Each Ti implant was incubated overnight under agitation at room temperature in 1 ml of the dopamine solution. After that, implants were washed five times with water until unbound dopamine was removed.

**Figure 1:**
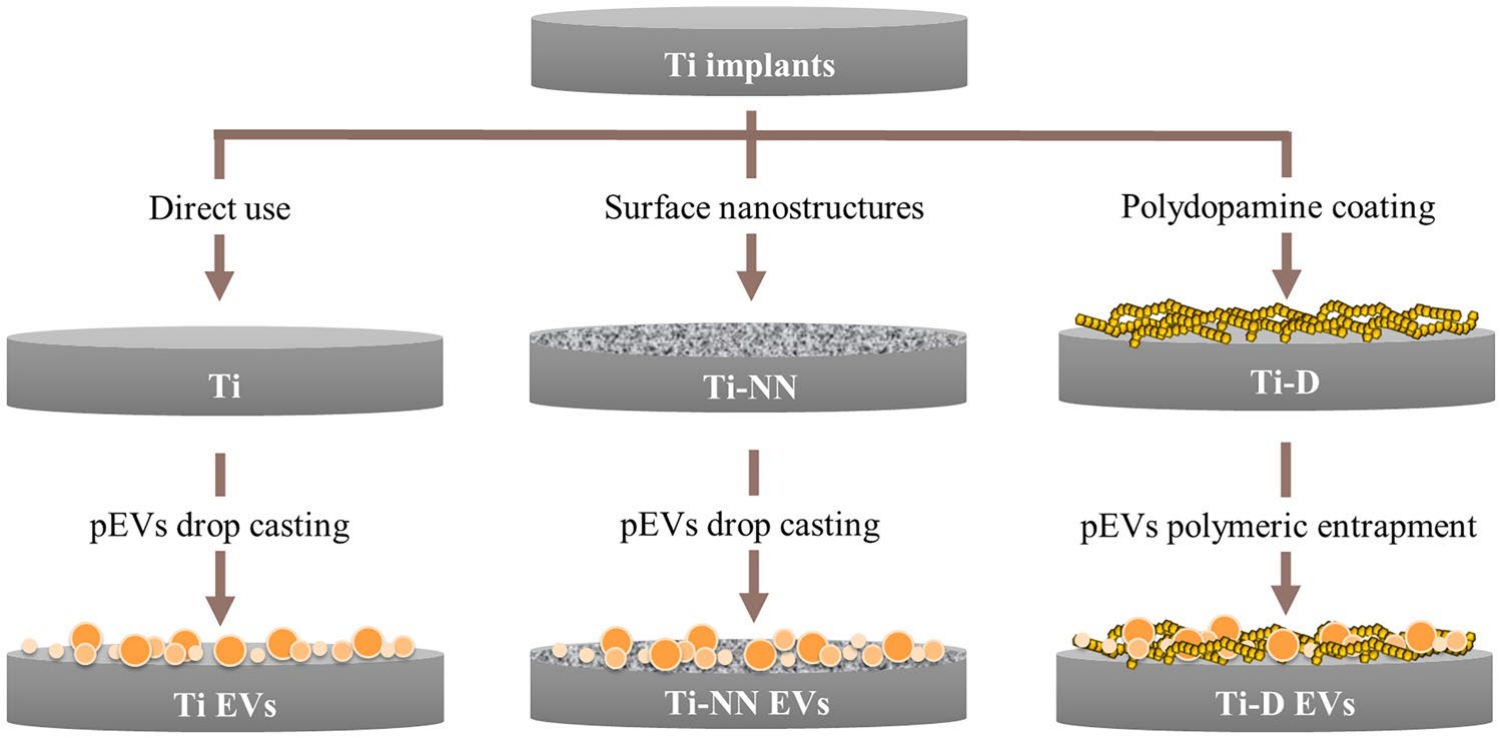
Implant functionalization strategies. Titanium discs were prepared for their direct use (Ti), nanostructuration (NN) or dopamine deposition (D). Coating was done by adding a pEVs solution to obtain Ti functionalized with EVs (Ti-EVs), NN functionalized with EVs (NN-EVs) or D functionalized with EVs (D-EVs).

After surface preparation, a 40 µl drop of pEVs containing 4 × 10^11^ EVs was incubated onto each surface at 37 °C for 2 h under vacuum conditions to induce their physisorption to the surface. Control groups, incubated with the same volume of PBS were prepared in parallel. Therefore, the different groups of implants prepared were direct titanium implants (Ti), titanium implants with EVs drop casting (Ti-EVs), nanonet structured titanium (NN), nanonet structured titanium with EVs (NN-EVs), dopamine covered titanium (D) and dopamine covered titanium with entrapped EVs (D-EVs) (Fig. 1).

### Scanning electron microscopy (SEM)

The implant surface morphology was analyzed using SEM (Hitachi S-3400N, Krefeld, Germany) after gold sputter-coating for non-EV functionalized samples. Images were acquired using secondary electrons, and 15 kV of voltage at × 1k and × 20k augments.

### Contact angle (CA)

The static contact angle was determined by the sessile drop method using a Nikon D3300 (AF-P DX 18–55 mm lent; Nikon, Tokyo, Japan) and analyzed with ImageJ software (National Institutes of Health, Bethesda, MD, USA). The contact angle measurements were performed using three samples of each non-EV functionalized group. A 1 µl ultrapure water drop was used as wetting agent.

### Cell culture

Human Mesenchymal Stem Cells derived from umbilical cord donors (hUC-MSC) were obtained from the IdISBa Biobank as approved by its Ethics Committee (IB1955/12BIO). hUC-MSCs were cultured at 37 °C and 5% CO_2_, using DMEM–low glucose (Gibco, Amarillo, USA) with 100 µg/ml penicillin–streptomycin (Biowest) and 20% certified foetal bovine serum (FBS, Gibco). Half of the medium was changed every 3 days.

For treatment, 10,000 cells were seeded on each implant surface in DMEM Low Glucose, 100 µg/ml penicillin–streptomycin and 1% FBS-EV’s depleted by centrifugation at 120,000×*g* for 18 h at 4 °C. Medium was refreshed every 3 days.

### Confocal laser scanning microscopy

PKH26 (Sigma) labelled EVs^32^ were used for surface functionalization following the same protocol described above. Ti surfaces were analyzed by confocal microscopy (Leica DMI 4000B equipped with Leica TCS SPE laser system, Wetzlar, Germany). Surfaces coated with PKH26 stained PBS were used as negative control (Supplementary Fig. II).

Alternatively, after 48 h of cell growth non-labelled EVs functionalized surfaces were stained with Phalloidin-Fluorescein Isothiocyanate (FITC; Sigma, St. Louis, USA) for cytoskeleton visualization.

### Cell cytotoxicity

Cell media was collected after 48 h of cell culture. Lactate dehydrogenase (LDH) activity was measured with Cytotoxicity Detection kit (Roche Diagnostics, Manheim, Germany) following the manufacturer’s instructions. For cytotoxicity determination, 0.1% Triton-X100 treated wells were used as high control, 100% cell death, while plastic seeded wells were used as low control, 0% cell death.

### Metabolic activity

Cellular metabolic activity was evaluated after 48 h of cell culture. Presto Blue reagent (Life Technologies, Carlsbad, CA) was used during 1 h of reagent incubation time following manufacturer’s protocol.

### NTA EVs release

Ti-EVs, NN-EVs and D-EVs were incubated with PBS at 37 °C for 14 days. At different time points (2, 6, 10 and 14 days) conditioned PBS was stored at – 80 °C and refreshed with new PBS. Stored PBS was analyzed through NTA. Particle concentration and size distribution were determined with Nanosight NS300 (Malvern Instruments, Malvern, UK).

### Alkaline phosphatase (ALP) activity

Cellular ALP activity was measured after 14 days of cell growth. Cells were lysed with 0.1% Triton X-100 in PBS performing two freeze/thaw cycles in liquid nitrogen. In parallel, a standard curve was prepared using calf intestinal ALP (Promega, Madison, WI, USA). ALP activity was evaluated with *p*-Nitrophenyl Phosphate (Sigma-Aldrich), by measuring the end product that absorbs at λ = 405 nm, after 2 h of incubation at 37 °C.

### Calcium content determination

Stored lysed samples from the ALP activity assay were diluted 1:1 in 1 N HCl. Then, total Ca^2+^ content was evaluated by inductively coupled plasma atomic emission spectrometry (Optima5300DV, PerkinElmer, USA). Data were compared with the CaCl_2_ standard curve included in the assay and Ti group was set at 100%.

### Statistical analysis

Normality was evaluated through Shapiro–Wilk test. For parametric data, one-way ANOVA and DMS or Games-Howell as post-hoc were performed. For non-parametric data, U de Mann–Whitney test was performed. Results were considered statistically significant at p < 0.05. SPSS 27.0 program (SPSS Inc., Chicago, USA) was used.

## Results and discussion

### Surface functionalization and characterization

Different strategies for Ti functionalization with EVs derived from cell culture media can be found in the literature, including chemisorption^23,24^ and physisorption approaches^23,25–28^. Here, we used EVs from platelets, which show a great osteogenic potential^11,12^*,* and we did a parallel comparison of three different functionalization strategies. As shown in Fig. 1, Ti discs were either passivated, nanostructured through electrochemical anodization or passivated followed by dopamine polymerization before pEVs coating.

**Figure 2:**
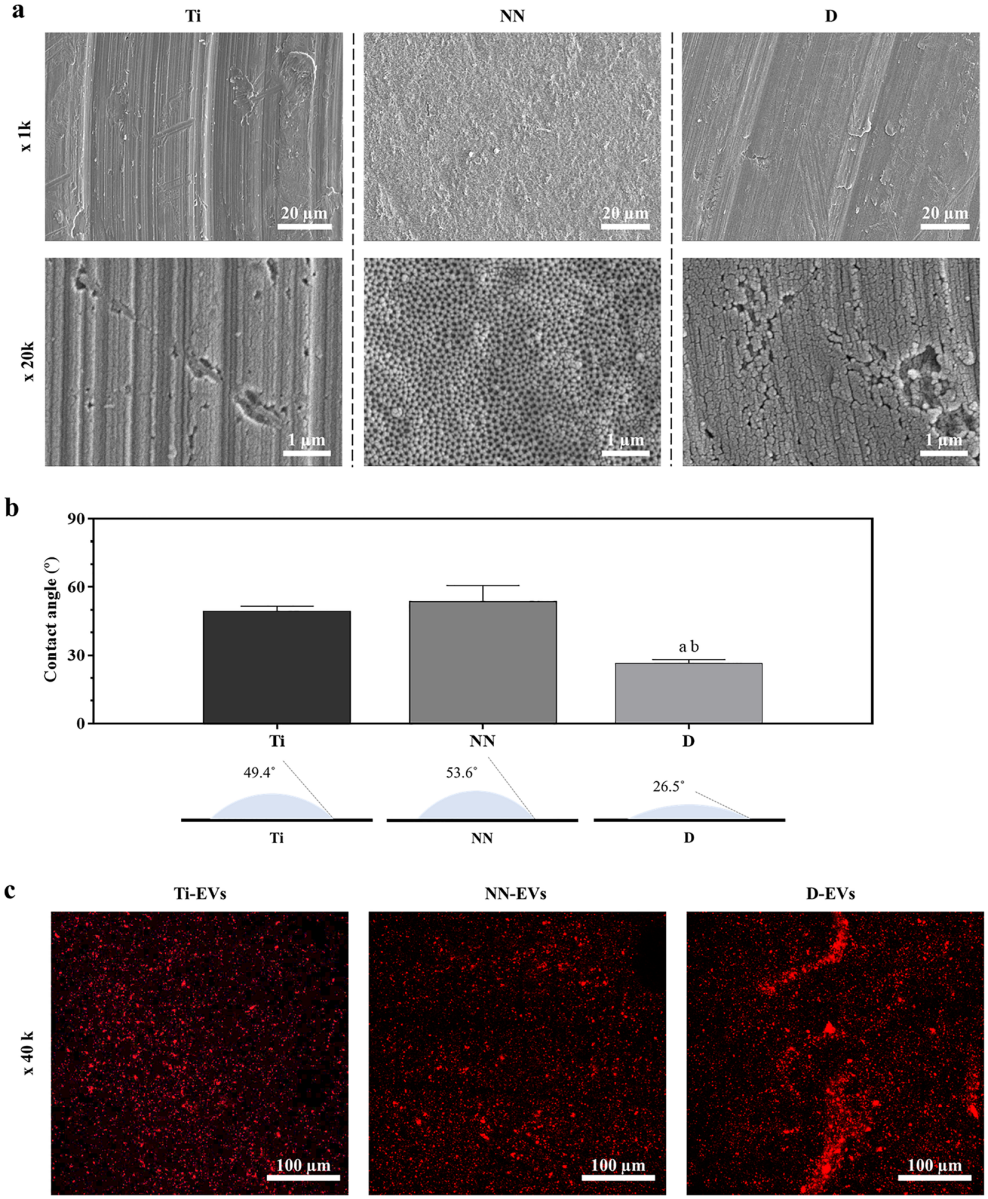
Titanium implant characterization. (**a**) Scaning electron microscopy images of Ti, NN and D surfaces at ×1000 and ×20,000. (**b**) Contact angle for Ti, NN and D surfaces using a 1 µl water drop (n = 3). Values represent the mean ± SEM. Data were compared by ANOVA, using Games-Howell as post hoc. Statistically significant differences were considered for p < 0.05 and represented with ^a^compared to Ti, ^b^compared to NN. (**c**) Confocal laser scanning microscopy images of surfaces functionalized with PKH26 stained pEVs (shown in red).

Surface topography and wettability may influence EV binding and release, and, consequently, modulate implant-cell interaction and performance. Prior to pEVs coating, Ti surface topografy was evaluated by Scanning Electron Microscopy (Fig. 2a) and its wettability by contact angle (CA) measurements (Fig. 2b). We could confirm nanostructuration of NN surfaces, being comparable to that of previous reports^31^. All surfaces were hydrophilic, with CA lower than 90°. Moreover, D implants presented significantly smaller CA measurements than Ti and NN groups, being the most hydrophilic surface.

Prior to functionalization, pEVs were labelled with the fluorescent dye PKH26, a fluorophore with a lipophilic carbocyanine that ancors into the lipid bilayer for fluorescence imaging^33,34^. This way, the presence of pEVs onto the different implant surfaces after functionalization through confocal laser microscopy could be evaluated. As shown in Fig. 2c, pEVs (red dots on the images) were bound to all the imaged surfaces. Evenmore, from a qualitative point of view, D-EVs present higher amount of binded EVs on their surface, that could be in part due to its higher hydrophilicity, facilitating the penetration of the pEV solution into the polymeric net on the surface. Moreover, polydopamine coating adhesive properties have been widely described^35^, also allowing higher binding of the pEVs onto the surface. In contrast, Ti-EV and NN-EV presented a more homogeneus distribution over the surface than D-EVs. Indeed, polydopamine formation and deposition depends on pH conditions, time or presence of catalic agents^35^, showing more hetereogeneity than the other evaluated surfaces.

### Extracellular vesicle release

**Figure 3:**
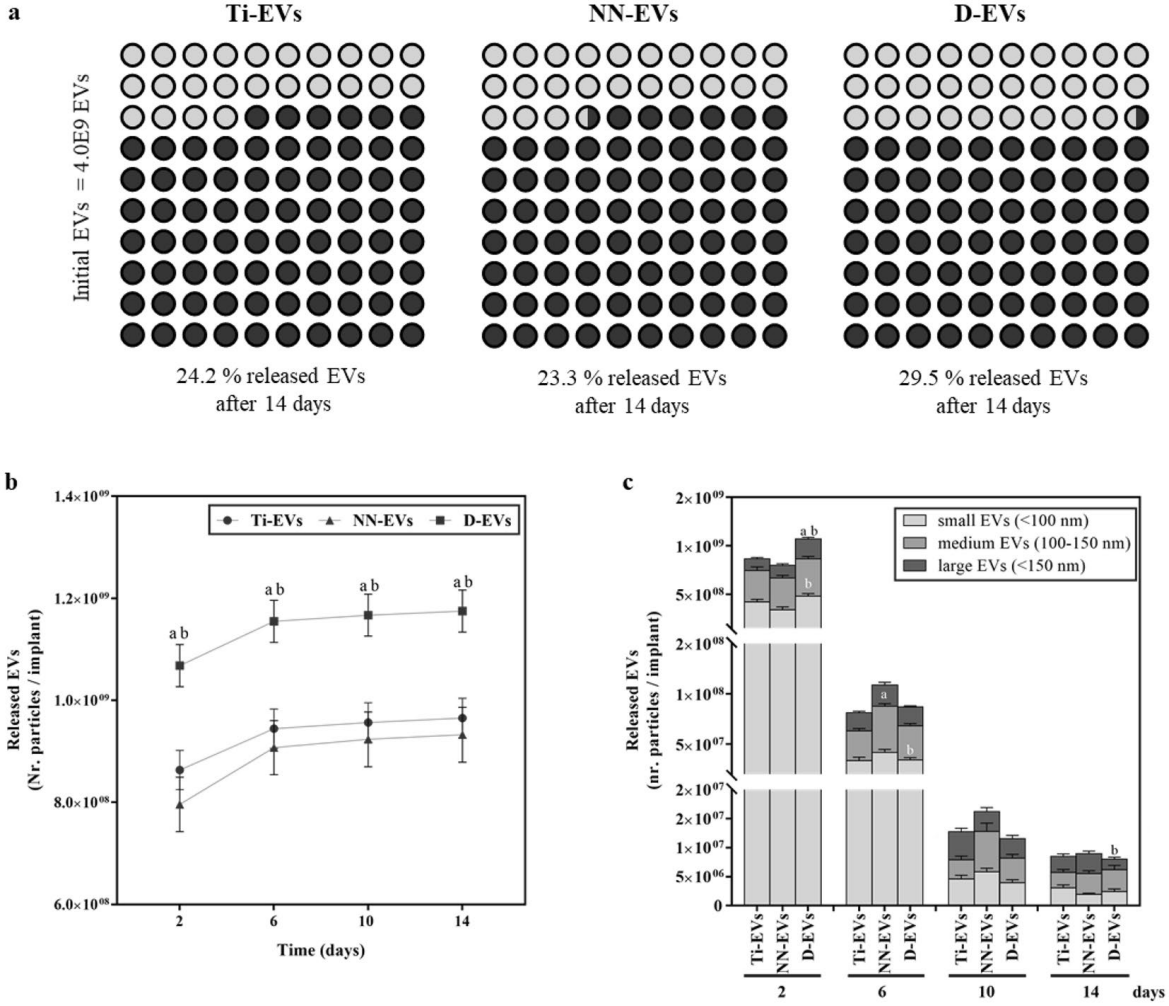
pEVs release over time. (**a**) Percentage of released EVs after 14 days for Ti-EVs, NN-EVs and D-EVs. (**b**) Accumulative pEVs release for each functionalized implant measured after 2, 6, 10 and 14 days. (**c**) Released pEVs according to size at 2, 6, 10 and 14 days. Small EVs (pale gray) correspond to sizes smaller than 100 nm, medium EVs (gray) are between 100 and 150 nm and large EVs (dark gray) are larger than 150 nm. Values represent the mean ± SEM and they were compared by ANOVA, using DMS as post hoc for data of 2 days and 10 days, and Games-Howell as post hoc for data of 6 days and 14 days. Three different samples were evaluated per group and statistically significant differences were considered for p < 0.05 and represented with ^a^compared to Ti-EVs and ^b^compared to NN-EVs.

Along with the different pEVs binding capacity onto the different evaluated surfaces, we were specially keen on EVs release profile from the implants. Thus, a quantitative release study was conducted during 14 days for each group, analysisng the released EVs after 2, 6, 10 and 14 days of PBS incubation (Fig. 3). After 14 days of incubation, the total initial amount of loaded EVs was not released from any of the evaluated surfaces, being 24.2 ± 1.0% for Ti-EV, 23.3 ± 1.4% for NN-EV, and 29.5 ± 1.3% for D-EV (Fig. 3a).

Further evaluation confirmed a gradual release over time (Fig. 3b). Though, the accumulative release also suggests that there is a burst release during the first 6 days and then, the number of EVs released per day decreases at each observed time point, as expected. At day 14, only a small number of EVs was released, despite the possibility of an uncomplete release, it is also possible that EVs suffer aggregation or fusion onto the Ti surface or even get disrupted and lose their vesicular structure due to the functionalization process^36,37^. These phenomena could explain why not all the initial EVs can be recovered after the 14 days of study. Nevertheless, further experiments should be performed to assess this possibility, tough the vesicular structure at 48 h is preserved and vesicle-like structures can be detected through TEM images (Supplementary Fig. III). Moreover, it is possible that the release starts at earlier time points than 48 h, but still, the evaluated time points were selected in agreement to the cell culture functional experiments detailed in the next section, since all the released EVs from the implant surface can be sensed by cells until 48 h, that is until the first media change.

In addition to the sustained release results, in all the evaluated time points, D-EV presents a significantly higher amount of released EVs compared to Ti-EV and NN-EV. This is in line with the higher pEVs binding capacity of this surface (Fig. 2c), probably due to polydopamine adhesive properties^35^. Therefore, it seems that dopamine polymeric entrapment is the most efficient strategy for pEVs functionalization onto Ti surfaces.

Keeping in mind that EVs are a heterogeneous population with different sizes and cargos, each probably delivers different messages to the receptor cells. We performed a more detailed analysis of the released EVs per day, since a different release profile may led to a different response induction^38^. For this reason, EVs were classified into small EVs (less than 100 nm), medium EVs (between 100 and 150 nm) and large EVs (bigger than 150 nm), as according to size EVs can present differences in their functional effects. After 2 days, D-EVs had released a significantly higher amount of small EVs than NN-EVs surfaces and also a significantly higher amount of large EVs than Ti-EVs and NN-EVs surfaces (Fig. 3c). This result is in line with the fact that D-EVs present an overall higher release than the other two surfaces. Then, from day 6, NN-EVs surfaces released a significantly higher amount of medium size EVs than the other surfaces. A similar pattern is shown on the EVs released at day 10. Finally, EVs released at day 14 from D-EVs surfaces presented a significantly lower number of large EVs compared to Ti-EVs and NN-EVs.

These results support the idea that the size profile of the released EVs depends on time and the surface functionalization strategy followed. For all the evaluated surfaces, the release of small EVs decrease over time, being, small EVs, mostly released at the earlier time points. However, each surface presented a different release profile. For instance, Ti-EVs showed an increased release of large EVs over time, while for NN-EVs surface, the release of the medium size EVs proportion remained nearly constant over time. Besides, D-EVs showed subtile changes on the size distribution of the released medium and large EVs over time.

Thus, the interactions established between the EVs and the surfaces may influence these changes of size in the released EVs. For Ti surfaces, only physisorption and fable physical interactions are established. The release is mainly driven by buoyant forces in which small EVs are easily disattached compared to medium or large EVs, because Van der Waals interactions are easily overcome^37^. This process may also be applicable for NN and D, despite the existence of other factors.

As regards to NN-EV, this surface presents a nanostructuration with a periodical arrangement which may interact preferently with specific pEV sizes, explaining the release profile of medium size EVs. Actually, some studies suggest that nanoarrays interact differently depending on the size of the array and the vesicular body^39^. For D-EV, this surface consisted of a polymeric entrapment which retains all the EVs, hindering their diffusion and avoiding an early release of most of the small EVs. Thus, although D-EV still presents a first release of small pEVs, it is not as overstated as in Ti-EV because of the adhesive effect of polydopamine^35^, which may be the main retaining force and it is not as weak as mear physisorption. In consequence, D may retain more efficiently all pEVs but it still allows the smaller ones to diffuse easily through the polymeric layer.

Overall and independently of the reason behind the pEVs release profile shifts, it is important to take into consideration this phenomena because different pEV populations may present different functionality and applications^38^. Therefore, functionalization strategies should be chosen carefully knowing that they may alter the EV population that preferently attacks to the surface and the release profile of EVs according to size. Nonetheless, further work evaluating the cargo of EVs in each release profile could shed some light in the molecular mechanism underlying this different functionality.

### Mesenchymal stem cell functional study

**Figure 4:**
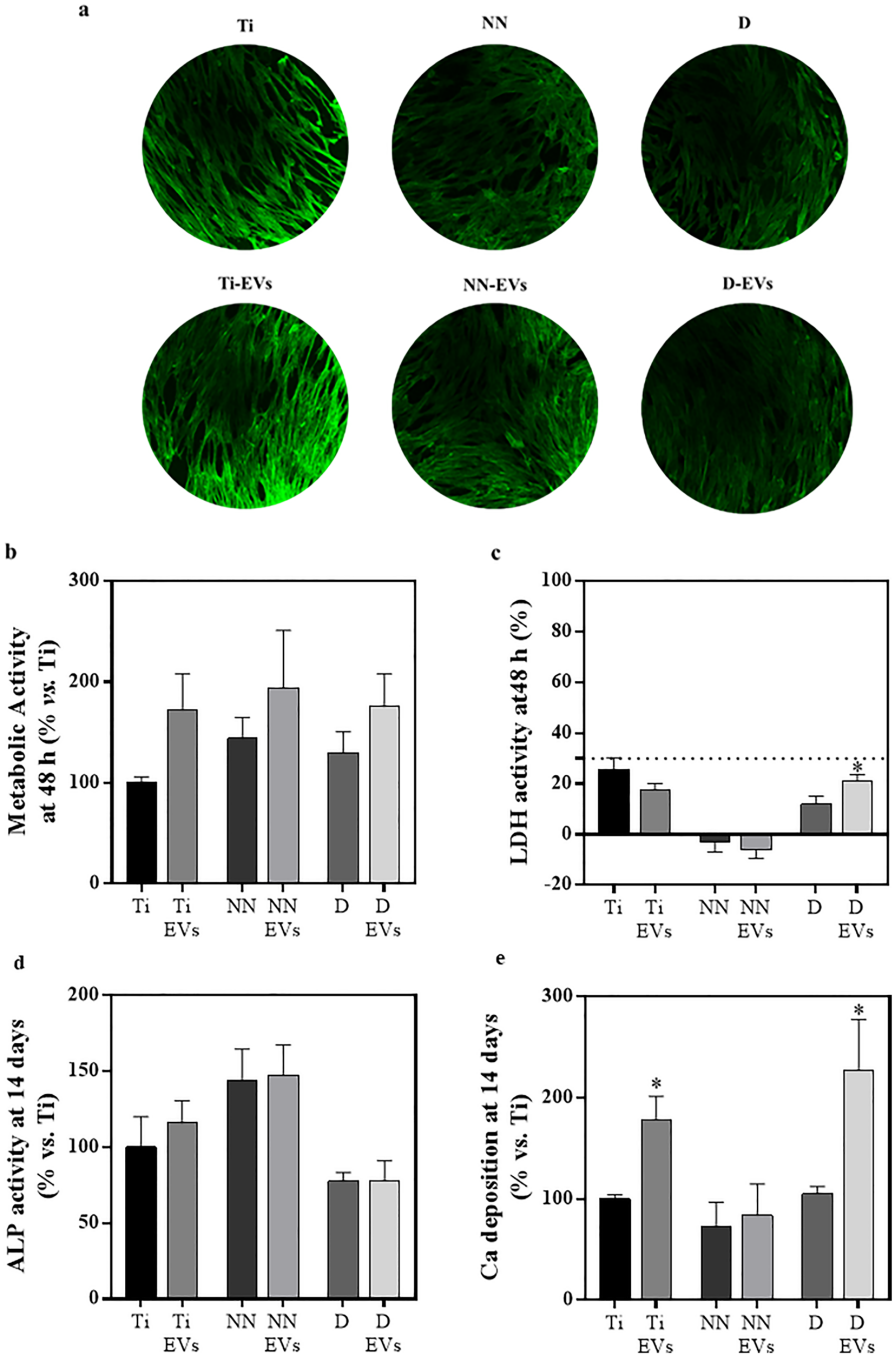
In vitro biocompatibility and MSC osteogenic differentiation. (**a**) Confocal images of implants surface after 48 h of MSC growth. Green signal corresponds to actin filaments. (**b**) Metabolic activity after 48 h of treatment, data of Ti group was set as 100%. (**c**) LDH activity measured in cell culture media after 48 h of cell growth. Cells seeded on TCP were set as 0% while cells treated with triton were set as 100% of LDH activity. A dashed line is added at 30% of LDH activity. (**d**) ALP activity after 14 days of cell culture. Ti ALP activity is set as 100%. (**e**) Ca deposition after 14 days of cell growth, Ca^2+^ levels of Ti were set as 100%. Three independent experiments were performed, each of them with three different samples for Ti, Ti-EVs, D and D-EVs (n = 9), while only two independent experiments were performed for NN and NN-EVs (n = 6). Values represent the mean ± SEM. Metabolic activity, LDH activity and Ca deposition were compared with t-test comparisons. Statistically significant differences were considered for p < 0.05 and represented with *compared to the respective control group without EV coating.

Then, an in vitro functional study was performed to test the effects of the different implant surfaces on biocompatibility and cell differentiation (Fig. 4). First, after 48 h of cell growth onto the surfaces, biocompatibility was assessed through confocal microscopy (Fig. 4a), metabolic activity (Fig. 4b) and cytotoxicity levels, through the determination of LDH activity released to cell culture media (Fig. 4c). Cells cultured onto all the different surface groups showed a positive signal of actin filaments (green). All pictures were taken under the same optical conditions, although Ti and Ti-EV present a more intense color. This difference may be caused by the light interactions induced by the different interfaces, as nanostructures or polymeric depositions^40^. Moreover, metabolic activity at 48 h, despite not reaching statistical significance, presented increased values for EV functionalized groups respect the non-functionalized surfaces, while all groups presented LDH levels under 30%. These results suggest a normal cell growth and no cytotoxic effects, while for all cases pEV functionalization indicates an improvement on cellular activity, according to metabolic activity results.

After 14 days, ALP activity (Fig. 4d) and Ca^2+^ deposition content (Fig. 4e) were determined. D and D-EVs show a decrease in ALP activity compared to NN and NN-EV surfaces. On the other hand, Ti-EV and D-EV presented higher Ca^2+^ deposition levels compared to Ti, NN and D. ALP is a biochemical osteogenic marker related to extracellular matrix calcium mineralization and osteoblast activity. It has been shown that ALP levels decrease, once mineralization stage starts^41^, explaining why D-EVs present high Ca^2+^ and low ALP levels. Therefore, these results corroborate the previously shown osteogenic effect of pEVs^11,12^, since we observed increased differentiation towards the osteogenic lineage of hUC-MSCs without the addition of osteogenic supplements. However, depending on the surface and the functionalization strategy followed for Ti functionalization, pEVs effects vary in intensity.

On the one hand, ALP activity shows no statistical difference attributed to pEV effect. However, bare functionalization strategies are responsible for differences between groups, for instance D and NN; or, for Ca^2+^ deposition levels, between D and Ti. Therefore, if an osteogenic effect is desired, it is important to use a surface modification which enables this effect. On the other hand, in terms of Ca^2+^ deposition, it seems that the combination with pEVs can boost the osteogenic effects for some strategies such as bare Ti functionalization and D polymeric entrapment while not for other strategies like nanostructuration. These differential biological effects found for the different pEV functionalized surfaces might be related with the fact that not all the evaluated surfaces are able to adhere and release the same amount nor the same kind of size subpopulation of pEVs; for instance, middle sized pEVs are the most abundant released from NN-EVs.

Overall, physisorption approaches for Ti functionalization with EVs must consider analyzing the preferent subpopulation release. Physical strategies enhance the adhesion and release of certain EVs according to their physical parameters as we have observed in this study. A possible alternative that some researchers have followed are chemisorption approaches^23,24^. However, it is possible that in this case the functionalization is driven specifically by certain kind of chemical groups present in the EVs and ending with only a specific subpopulation according to biochemical properties instead of physical characteristics. In any case, the functionalization strategy is an important fact that may alter EVs functional effects and must be evaluated before claiming the initial properties attributed to EVs.

Furthermore, different EVs sources have been evaluated for Ti functionalization, including macrophages^26^ or stem cells^24,25,27^. In those studies, osteogenic capabilities were observed, however, the fact of using blood derived EVs, like pEVs, allows an easier manipulation, avoiding cell culture and easing a future clinical use^6^. Some studies also introduced complex settings such as the BMP2 stimulation of cells^26^ or the chemical modification of EVs^23,24^. However, before being able to translocate such elaborated approaches it is necessary to understand the EVs biology and their action path, because EVs are heterogeneous samples which may have a variety of effects depending on each subpopulation.

## Conclusions

Our results demonstrate the impact of the functionalization strategy followed for EVs coating on the resulting biological effect. EVs have shown high physicochemical stability and biocompatibility as well as numerous therapeutic effects. In addition, EVs can be modified through different technologies for uncounted applications. Biomaterial functionalization with naturally derived or technologically modified EVs is an emerging research field that is rapidly growing. However, EVs are a heterogeneous population, which makes them more sensitive to differences on the functionalization strategy used.

We have shown, how the different strategies followed for Ti functionalization with pEVs modify the release profile of different EVs subpopulations, leading to a different biological response. Thus, the increased osteogenic effect shown by pEV used in solution was maintained in Ti surfaces functionalized with EVs after passivation and through dopamine entrapment, while this effect was not observed after pEV functionalization of nanostructured surfaces.

## Supplementary Information

Supplementary Tables.

Supplementary Figures.

## Data Availability

Data generated or analyzed during this study are included in this published article as a supplementary file.

## Abbreviations

ALP: Alkaline phosphatase
CA: Contact angle
D: Dopamine coated implants
D-EVs: Dopamine coated implants functionalized with extracellular vesicles
EV: Extracellular vesicle
FBS: Foetal bovine serum
LDH: Lactate dehydrogenase
hUC-MSC: Mesenchymal stem cell from human umbilical cord
NN: Nanonets implants
NN-EVs: Nanonets implants functionalized with extracellular vesicles
NTA: Nanoparticle tracking analysis
PBS: Phosphate buffer solution
pEV: Platelet derived extracellular vesicle
PL: Platelet lysate
PRP: Platelet rich plasma
SEM: Scaning electron microscopy
Ti: Titanium implants
Ti-EVs: Titanium implants functionalized with extracellular vesicles
