# Supplementary material for: Customizing the extracellular vesicles release and effect by strategizing surface functionalization of titanium
Source: Sci Rep. 2022 May 5;12:7399. doi: 10.1038/s41598-022-11475-3 (PMC9072683; doi:10.1038/s41598-022-11475-3)
Supplement: Supplementary file 2 — Supplementary Figures. [file 41598_2022_11475_MOESM2_ESM.docx]

Supplementary data


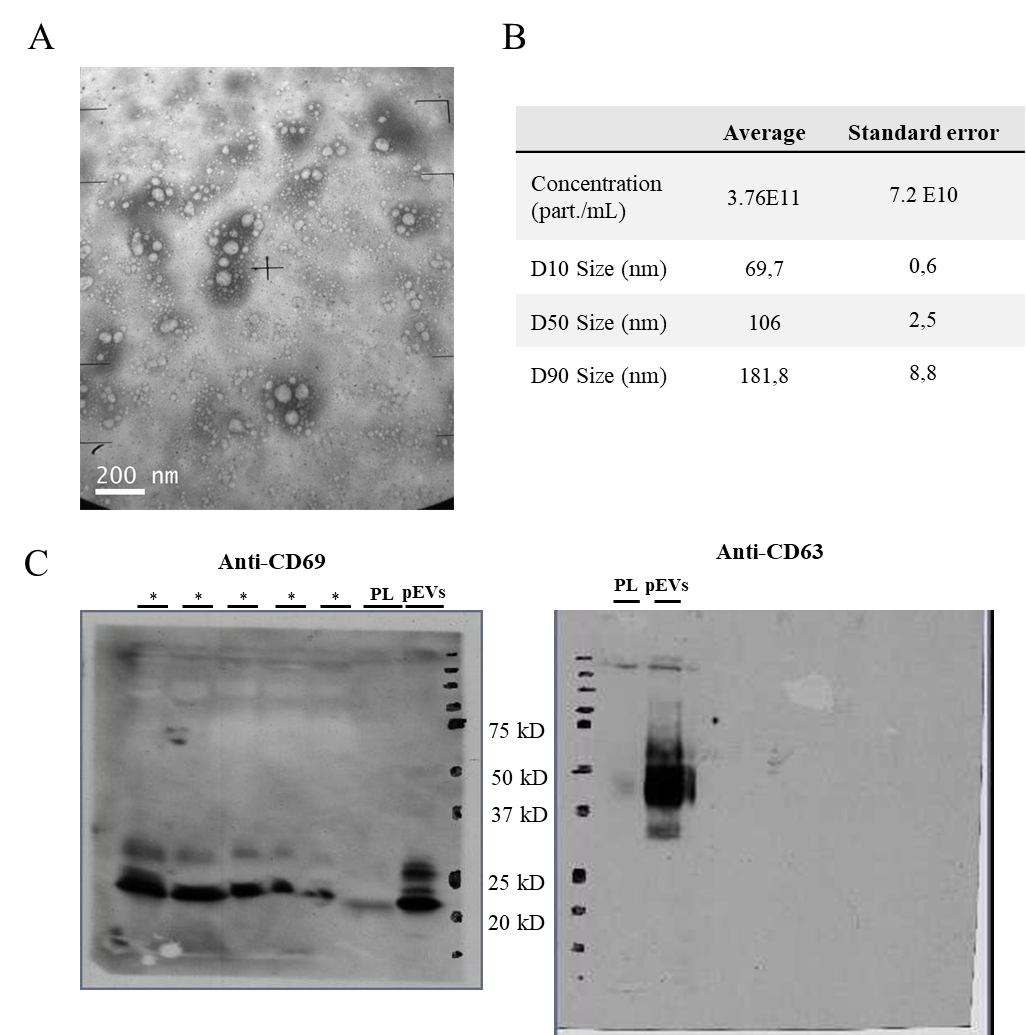


**Supplementary Figure I. Platelet Extracellular Vesicle (pEVs) characterization.** (**A**) Wide-field transmission electron microscopy image of pEVs taken at x50 k augments. (**B**) Particle concentration and size value of deciles 10 (D10) decile 50 (D50) and decile 90 (D90). (**C**) Presence of CD9 and CD63 for PL and pEVs. Full-length gels are shown and PL and pEVs wells are indicated, while * stands for samples not related to this work but that were loaded in the same gel. The same amount of protein was loaded per well (5 µg).


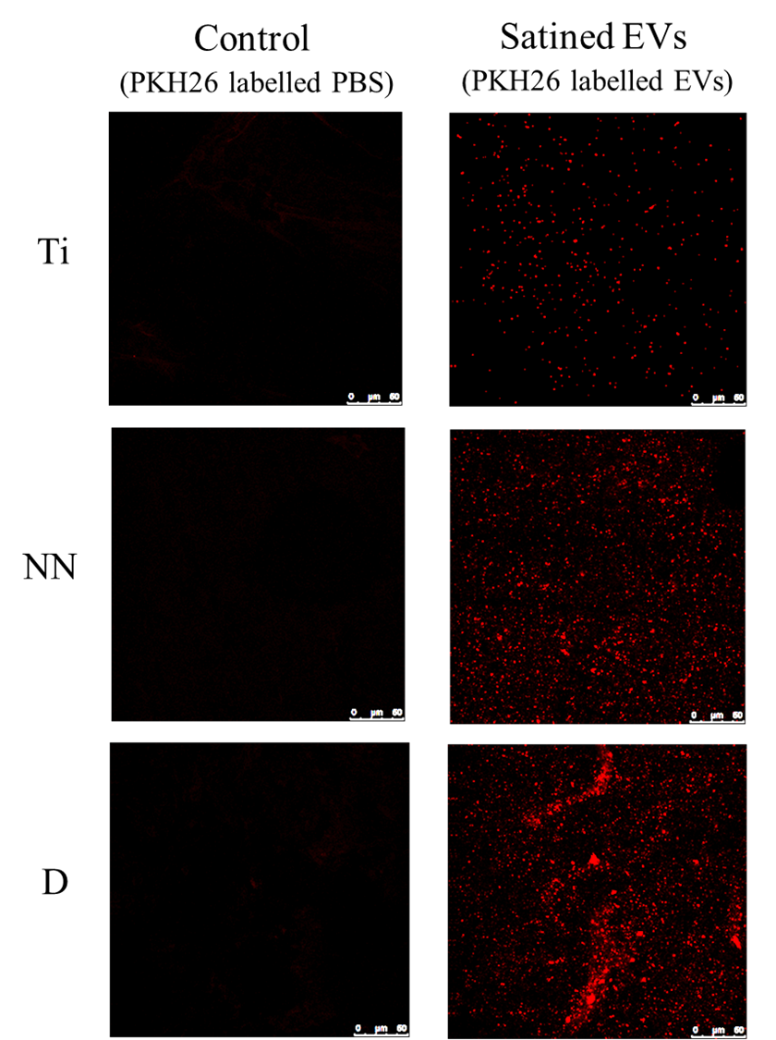


**Supplementary Figure II. Confocal laser scanning microscopy images of surfaces.** Representative images of TI, NN and D surfaces with PKH26 stained PBS (Control) and stained EVs.


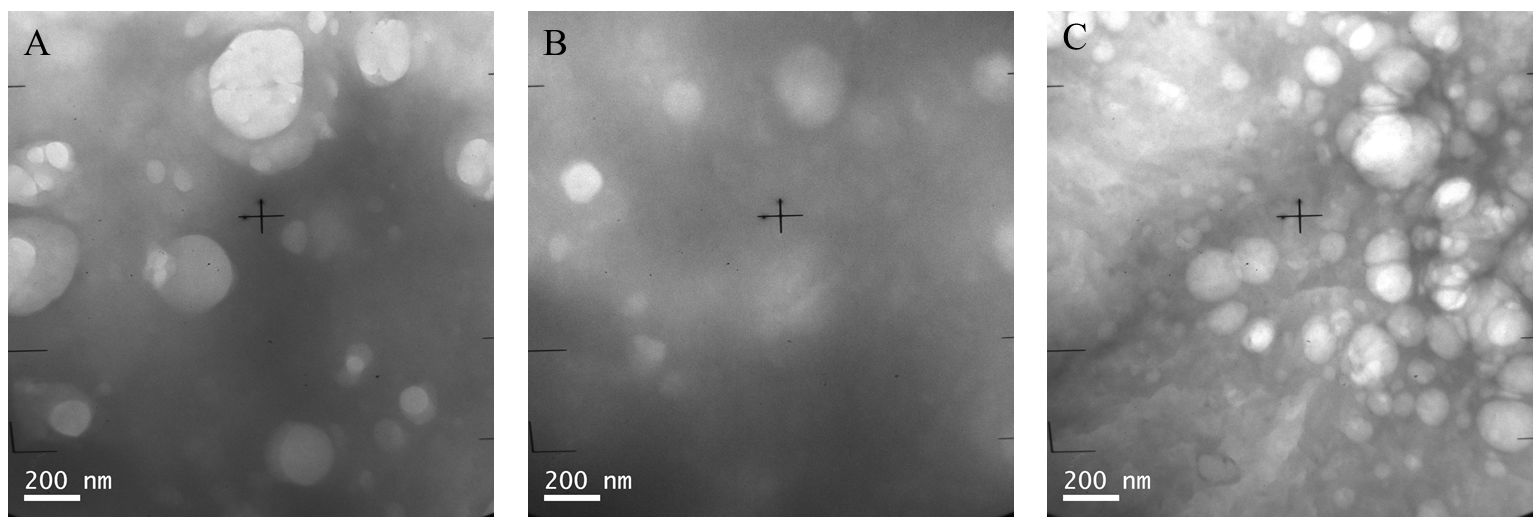


**Supplementary Figure III. TEM images of pEVs released after 48 h of PBS incubation.** (**A**) pEVs released by Ti-EV surfaces. (**B**) pEVs released by NN-EV surfaces. (**C**) pEVs released by D-EV surfaces. All images were taken at x50 k augments.
